# Heterologous Expression and Catalytic Properties of Codon-Optimized Small-Sized Bromelain from MD2 Pineapple

**DOI:** 10.3390/molecules27186031

**Published:** 2022-09-16

**Authors:** Rafida Razali, Fikran Aranda Fahrudin, Vijay Kumar Subbiah, Kazufumi Takano, Cahyo Budiman

**Affiliations:** 1 Biotechnology Research Institute, Universiti Malaysia Sabah, Kota Kinabalu 88400, Sabah, Malaysia; 2 Department of Biomolecular Chemistry, Kyoto Prefectural University, Hangi-cho, Shimogamo, Sakyo-ku, Kyoto 606-8522, Japan

**Keywords:** bromelain, expression, purification, catalytic activity, metal ion, antioxidant

## Abstract

Bromelain is a unique enzyme-based bioactive complex containing a mixture of cysteine proteases specifically found in the stems and fruits of pineapple (*Ananas comosus*) with a wide range of applications. MD2 pineapple harbors a gene encoding a small bromelain cysteine protease with the size of about 19 kDa, which might possess unique properties compared to the other cysteine protease bromelain. This study aims to determine the expressibility and catalytic properties of small-sized (19 kDa) bromelain from MD2 pineapple (MD2-SBro). Accordingly, the gene encoding MD2-SBro was firstly optimized in its codon profile, synthesized, and inserted into the pGS-21a vector. The insolubly expressed MD2-SBro was then resolubilized and refolded using urea treatment, followed by purification by glutathione S-transferase (GST) affinity chromatography, yielding 14 mg of pure MD2-SBro from 1 L of culture. The specific activity and catalytic efficiency (*k_cat_*/K_m_) of MD2-SBro were 3.56 ± 0.08 U mg^−1^ and 4.75 ± 0.23 × 10^−3^ µM^−1^ s^−1^, respectively, where optimally active at 50 °C and pH 8.0, and modulated by divalent ions. The MD2-SBro also exhibited the ability to scavenge the 2,2-diphenyl-1-picryl-hydrazyl-hydrate (DPPH) with an IC_50_ of 0.022 mg mL^−1^. Altogether, this study provides the production feasibility of active and functional MD2-Bro as a bioactive compound.

## 1. Introduction

Bromelain is a member of the papain family that contains a complex and diverse natural mixture of proteases. It belongs to the family of sulfhydryl proteolytic enzymes and has a catalytic mechanism that involves the triad Cys-His-Asn/Glu [1,2,3]. This enzyme can be found in the pineapple plant (*Ananas comosus*). Depending on the source, it is usually classified as either fruit bromelain or stem bromelain. Apart from the stem and fruits, bromelain was also reported to be present in pineapple peel, core, crown, and leaves [4]. While bromelain is a unique cysteine protease name for pineapple, other cysteine proteases are widely distributed in plants and animals, including papain from papaya (*Carica papaya*) [5] and ficin from *Ficus insipida* [6]. In addition, cysteine protease was also found in viruses [7].

Bromelain possesses significant and notable therapeutic properties such as anti-inflammatory, anti-thrombotic and fibrinolytic effects, inhibition of platelet aggregation, anti-cancer activity, immunomodulatory effects, enhanced wound healing and adsorption of drugs, particularly antibiotics, and cardiovascular and circulatory improvement [8,9]. It is also widely used in the food industry and considered a food supplement approved by the Food and Drug Administration of the United States of America, and is now freely available in the market [1,10]. It can be absorbed into the human intestines without degradation or losing biological activity [11,12,13]. Moreover, bromelain is also known to have the ability to hydrolyze meat proteins, particularly myofibril and connective fractions, and is considered a meat tenderizer that can be used traditionally [14].

Earlier, the whole-genome sequence of MD2 pineapple revealed the presence of 14 genes encoding cysteine proteases under the bromelain protease group [15]. Interestingly, these 14 genes encode various molecular weights of cysteine proteases ranging from 19 kDa to more than 200 kDa [15,16]. However, most of the studies on cysteine proteases of bromelain deal with medium-sized bromelain with a size of about 20–40 kDa [1,17,18,19]. So far, no study has reported on small-sized bromelain (20 kDa or less). Production of single cysteine protease bromelain for further applications as an enzyme-based bioactive compound is challenging due to a lengthy purification process. The use of the recombinant approach to producing active bromelain from a single gene is feasible yet challenging due to its solubility issue [1].

Our previous in silico study showed that the small-sized bromelain of MD2 pineapple (MD2-SBro) exhibited different structural features than the medium-sized bromelain of MD2 pineapple (MD2-MBro). Structurally, the Cys catalytic site of MD2-SBro is found to be located at the flexible loop, which is quite mobile and affects its proximity to the substrate [16]. In addition, both MD2-SBro and MD2-MBro also displayed differences in the hydrophobicity of the substrate-binding cavity. Earlier, we demonstrated that recombinant MD2-MBro produced under *Escherichia coli* (*E. coli)* was catalytically active with the specific activity and catalytic efficiency of 6.13 ± 0.01 U mg^−1^ and 5.64 ± 0.02 × 10^–2^ μM^−1^ s^−1^, respectively [1].

This report provides the first experimental evidence on catalytic properties of recombinant MD2-SBro produced from *E. coli* host cells. We demonstrated that MD2-SBro was catalytically active with the activity modulated by pH, temperature, and metal ions. In addition, the antioxidant activity of this protein was also detectable.

## 2. Materials and Methods

### 2.1. Gene Optimization, Synthesis and Expression System Construction

The MD2-Sbro gene sequence was retrieved from NCBI with accession number OAY85828.1. The gene sequence was then optimized using OptimumGene^TM^ (Piscataway, NJ, USA) according to the codon usage preference of *E. coli*, and chemically synthesized under the GenScript outsource service (Piscataway, NJ, USA). The gene was provided in the pUC18 plasmid, designated as pUC18-SBro. Further, to construct the expression system for the gene, the SBro gene was amplified from the pUC18 plasmid using polymerase chain reaction (PCR) with a pair of specific primers. The sequences of the PCR primers used are as follows: 5′-CGAAGCTTATGGCGGAGTACGGTCGTGTG-3’ (forward, with *Hind*III site) and 5′-GGCTCGAGGCCCCACCAGGAACCCCAGC-3’ (reverse, with *Xho*I site). PCR was performed using KOD polymerase (Toyobo Co., Ltd., Kyoto, Japan) with the GeneAmp PCR system 2400 (Applied Biosystems, Tokyo, Japan). The amplicon was then digested with restriction enzymes of *Hind*III and *Xho*I and ligated into the pGS-21a expression vector using the DNA Ligation Kit, Mighty Mix (Takara, Japan). The success of ligation was confirmed using an insert check with restriction enzymes and nucleotides using the Prism 310 DNA sequencer (Applied Biosystems). The recombinant DNA of the MD2-SBro gene and pGS-21a is designated as an expression system of pGS21-SBro. This expression system allows MD2-SBro to be expressed in a fusion form to a glutathione S-transferase (GST) tag at its N-terminal. This expression system was then transformed into *E. coli* BL21(DE3) using the heat shock method based on Froger and Hall [20].

### 2.2. Protein Expression

Expression of recombinant MD2-SBro was performed based on Razali et al. [1] with some modifications. Briefly, the transformed cells were cultured in Luria Bertani (LB) media supplemented with 100 µg mL^−1^ ampicillin and incubated at 37 °C at 180 rpm. The enzyme was induced by 1 mM IPTG (isopropyl β-D-1-thiogalactosidase (IPTG) once the OD_600_ reached 0.7, followed by a prolonged incubation at 37 °C, 180 rpm for 5 h. The culture was harvested by centrifugation at 8000× *g* for 10 min at 4 °C. The cell pellet was then washed twice and resuspended in 20 mM phosphate buffer, pH 8.0, containing 100 mM NaCl, followed by cell lysis by a sonication in ice. The soluble fraction was then separated from the cell debris (pellet) by centrifugation at 35,000× *g* for 30 min at 4 °C. Soluble and pellet fractions were aliquoted for protein expression and solubility checking under 15% SDS-PAGE (sodium dodecyl sulfate–polyacrylamide gel electrophoresis). The whole fractions were also kept for further steps.

### 2.3. Solubilization and Refolding of Insoluble Protein

For the protein expressed in an insoluble form, solubilization and refolding steps were performed using urea treatment according to the method described by Kannan et al. [21] and Yamaguchi and Miyazaki [22], with some modifications. Briefly, the inclusion bodies (pellets) obtained after sonication were resuspended in 20 mM phosphate buffer (pH 8.0) containing 8 M urea, 2 mM DTT, and 100 mM NaCl. The sample solution was incubated at 4 °C overnight, followed by centrifugation at 35,000× *g*, 4 °C for 30 min. The supernatant was then collected for dialysis to refold the protein by removing the urea against 20 mM phosphate buffer (pH 8.0) at 4 °C overnight. Following the dialysis, the sample was centrifuged at 20,000× *g* for 15 min. The supernatant was collected and considered as solubilized MD2-SBro in a crude form, which was then used for solubility checking under 15% SDS-PAGE and further purification steps.

### 2.4. Protein Purification

Purification of a crude form of solubilized MD2-Sbro was performed according to [23]. Briefly, the GSTrap HP 5 mL (GE Healthcare; Chicago, IL, USA) column was firstly equilibrated with the binding buffer (20 mM phosphate buffer, pH 8.0 containing 100 mM NaCl). The crude form of solubilized MD2-Sbro, which was filtered previously using a syringe filter 0.22 µM membrane (Pall Life Sciences; Port Washington, NY, USA), was then loaded into the column. The sample was then eluted by linear gradient at gradual increments from 0% to 100% of an elution buffer containing 50 mM Tris-HCl (pH 8.0) with 10 mM reduced glutathione. The presence and purity of eluted MD2-Sbro were then checked from the fractions across the peak of interest in the chromatogram using 15% SDS-PAGE. Finally, the fractions containing MD2-Sbro in acceptable purity were pooled and dialyzed against 50 mM Tris-HCl, pH 8.0.

The purified protein concentration was determined by the NanoDrop™ 2000 (Thermo Fisher Scientific; Waltham, MA, USA) on the basis that the absorbance at 280 nm of 0.1% (1 mg mL^−1^) solution is 1.69, as calculated based on Goodwin and Morton [24].

### 2.5. SDS-PAGE

The expression, solubility, and purity of the MD2-SBro protein were confirmed using 15% SDS-PAGE [25]. In addition, the gel was stained with Coomassie Brilliant Blue (CBB) dyes and visualized using a Gel Doc^TM^ XR+ imager (Biorad; Hercules, CA, USA).

### 2.6. Enzymatic Activity and Kinetic Parameters

The enzymatic activity of the purified protein was determined at 37 °C and pH 8.0 based on the method described by Razali et al. [1], with some modifications. It was determined using N-carbobenzoxyglycine *p*-nitrophenyl ester (N-CBZ-Gly-*p*NP) substrate [26]. Prior to adding the substrate, concentrations of the enzyme with different ranges (0.5–10 µg/mL) were incubated in 20 mM Tris-HCl buffer pH 8.0 at 37 °C for 5 min, with shaking to homogenize the solution. Then, the substrate with 50 µM of final concentration was added to the enzyme solution and incubated for 5 min. The product release was monitored at 340 nm using a Lambda 35 Perkin-Elmer UV-Vis spectrophotometer (Waltham, MA, USA). The amount of p-nitrophenyl (*p*NP) released was calculated based on an extinction coefficient for *p*NP of 6320 M^−1^ cm^−1^. One unit of the enzyme was defined as the amount of enzyme which produced 1 µmol of the product per minute. To determine the effect of GST on the specific activity of MD2-SBro, the specific activity of the protein was also observed in the absence or presence of free GST protein (Sigma Aldrich; St. Lois, MI, USA). The MD2-SBro was 10 µg/mL, while the concentration of free GST protein was prepared in 1:1, 1:5, and 1:50. The changes in the activity were then observed qualitatively based on the color changes due to the release of the *p*NP moiety.

Meanwhile, the kinetic parameters (V_max_ and K_m_) of MD2-SBro were calculated mathematically according to the Michaelis–Menten equation [27], using linear regression analysis through a double reciprocal (Lineweaver–Burk) plot [17]. The enzyme mixture in 20 mM Tris-HCl buffer pH 8.0 was first incubated at 37 °C for 5 min with shaking. The kinetic parameters were then determined against the substrate. Data were obtained by measuring the initial rate of hydrolysis by incubating the enzyme with different range concentrations of the substrate in 20 mM Tris-HCl buffer pH 8.0 at 37 °C. The maximal velocity (V_max_) and Michaelis constant (K_m_) were computed by plotting the data in GraphPad Prism 6 software. The assays were performed in triplicates, and data were shown as the mean ± standard deviation.

### 2.7. Optimum Temperature and pH

The optimum temperature for the catalytic activity of MD2-SBro was determined at different temperatures from 25–80 °C in 20 mM Tris-HCl buffer pH 8.0 with the detailed conditions described above. Meanwhile, the optimum pH of the protein activity was observed over the pH range of 4.0–10.0. For pH 4.0–6.0, it was measured in 20 mM citrate buffer, 20 mM Tris-HCl buffer for pH 6.0–8.0, and 20 mM Gly-NaOH for pH 8.0–10.0 [1]. The highest activity obtained during the measurement was set as 100% of the specific activity. The assays were performed in triplicates, and data were shown as the mean ± standard deviation.

### 2.8. Determination of EDTA and Metal Ions Effect

The influence of ethylenediaminetetraacetic acid (EDTA) and different metal ions (Ca^2+^, Cu^2+^, Mg^2+^, Zn^2+^, Ni^2+^) on the catalytic activity of MD2-SBro was performed according to the previous reports [1,28,29,30], with some modifications. The catalytic activity was examined by adding EDTA or different metal ions with a final concentration of 10 mM at the optimum temperature and pH [1].

### 2.9. Antioxidant Activity

The antioxidant activity was determined using a modified version of the free radical 2,2-diphenyl-1-picrylhydrazyl (DPPH) assay [31]. Various concentrations of proteins (0.05 mg/mL, 0.1 mg/mL, 0.2 mg/mL, and 0.5 mg/mL) were prepared in 20 mM Tris-HCl, pH 8.0. Then, 2.5 mL of MD2-SBro was mixed with 1 mL of DPPH and placed on ice for 30 min. The DPPH test for radical scavenging activity was monitored using a Lambda 35 Perkin-Elmer UV-Vis spectrophotometer (Waltham, MA, USA) at 517 nm. Ascorbic acid, which is known to exhibit antioxidant activity, was used as a positive control. The percentage values of radical scavenging activity by DPPH (A) were calculated using the formula below:

$$A\%=\frac{\mathrm{Abs}\;\mathrm{control}-\mathrm{Abs}\;\mathrm{sample}}{\mathrm{Abs}\;\mathrm{control}}\times 100$$

## 3. Results

### 3.1. Gene Optimization

In this study, the gene encoding bromelain MD2-SBro was optimized by increasing the GC content of MD2-SBro from 44.51% to 53.34%. In addition to the changes in GC content, the codon adaptation index (CAI) of the newly synthesized MD2-SBro was also adjusted from 0.40 to 0.95 (Table 1). All the changes were based on the preferences of *E. coli* as the host cells, according to Nussinov [32] and Akhtar et al. [33]. Further, the modification was only performed on the DNA level, where the amino acid sequence was not changed. Accordingly, the translated polypeptide from the optimized MD2-SBro gene is expected to have the same primary structure and fold into the same three-dimensional structure as the polypeptide from the original gene.

### 3.2. Protein Expression and Solubilization

**Figure 1:**
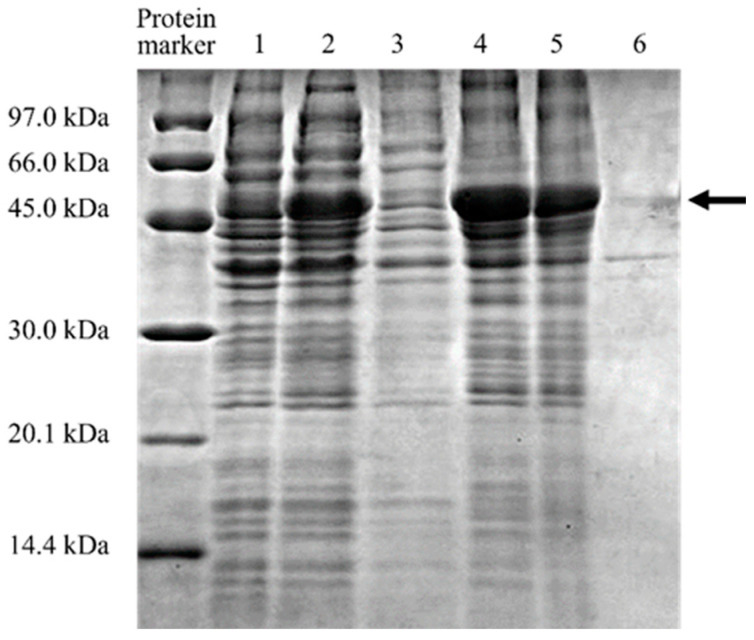
Expression and solubilization check of MD2-SBro protein. Lane 1: Before IPTG induction; Lane 2: After IPTG induction; Lane 3: Soluble fraction obtained after the sonication; Lane 4: Insoluble fraction obtained after the sonication; Lane 5: Soluble fraction obtained after the solubilization; Lane 6: Insoluble fraction obtained after the solubilization. The band corresponding to MD2-SBro is indicated by the arrow.

The optimized MD2-SBro was expressed in *E. coli* BL21(DE3), as indicated by the thick band with an apparent size of 50 kDa (Figure 1). This size is comparable to the theoretical size (calculated from the amino acid sequence) of MD2-SBro in a fusion form with a GST-tag, which is 50798.17 Da (GST-tag and linker = 30.32 kDa; MD2-SBro = 19.42 kDa). The 50 kDa band appeared only when IPTG induced the culture. Nevertheless, Figure 1 also showed that the 50 kDa band appeared in a pellet fraction after sonication, which indicated that MD2-SBro was expressed in an insoluble form (inclusion body). As the protein was expressed in an insoluble form, the solubilization and refolding were done using urea treatment, along with a reducing agent of DTT. The solubilized and refolded MD2-SBro was found to be in a soluble form, as shown by the appearance of a 50 kDa band in the soluble fraction after the treatment.

### 3.3. Protein Purification

**Figure 2:**
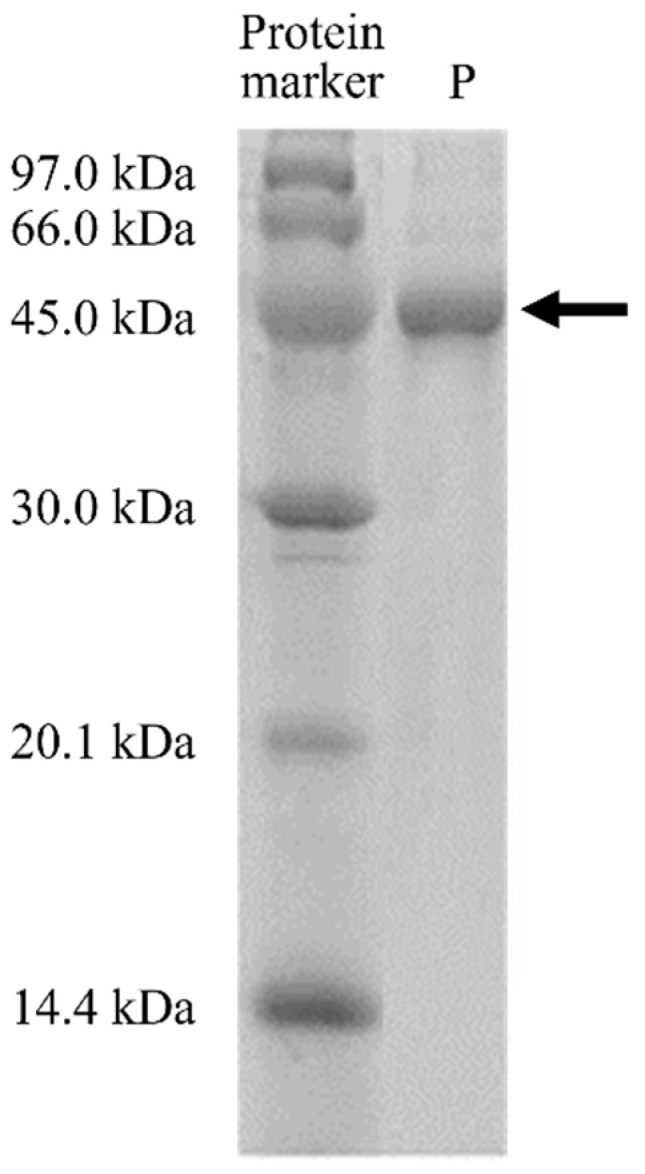
Purification check of MD2-SBro protein. Lane P: Purified protein.

The solubilized and refolded MD2-SBro was then purified by GST-affinity chromatography, resulting in a single 50 kDa band in 15% SDS-PAGE (Figure 2). The presence of the contaminants was undetectable under the gel, which showed that the MD2-SBro protein was successfully produced in high purity under single-step chromatography.

The detail of the purification profiles is shown in Table 2. The amount of purified MD2-SBro obtained from 1 L culture was 14 mg. Meanwhile, the enzymatic activity was calculated based on the amount of *p-*nitrophenol (*p*NP) released upon the digestion of the N-CBZ-Gly-*p*NP substrate. The release of the *p-*nitrophenol moiety is detectable as yellow color and quantitatively measurable by a UV-Vis spectrophotometer. Table 2 also showed that the specific activity of MD2-SBro was 3.56 ± 0.08 U/mg. Interestingly, the purification fold of MD2-SBro was found to be more than 40-fold.

**Figure 3:**
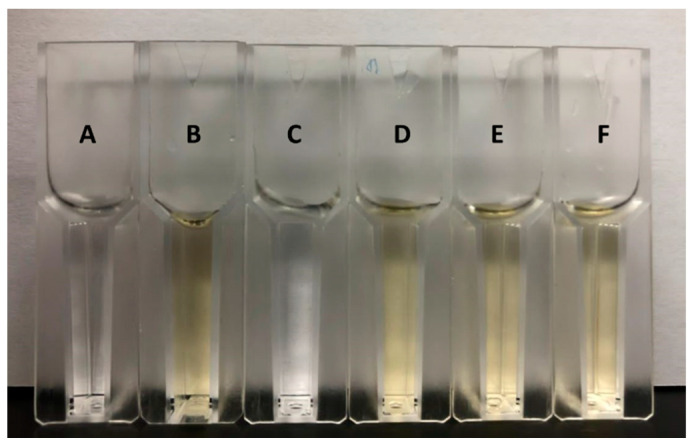
The proteolytic assay mixture of the N-CBZ-Gly-*p*NP substrate. Tube A is a blank (only substrate, without MD2-SBro and free GST protein). Tubes B and C are the mixture containing the substrate with MD2-Sbro and substrate with free GST protein, respectively. Tubes D, E, and F refer to the mixtures containing substrate, MD2-SBro, and free GST protein. The ratio of MD2-SBro and free GST protein were 1:1 (reaction D), 1:10 (reaction E), and 1:50 (reaction F).

Notably, when the activity of MD2-SBro was observed in the presence of free GST protein, the yellow color of the cocktail reaction was not changed by the addition of free GST protein. Meanwhile, no yellow color was detected when free GST protein was mixed with the substrate without MD2-SBro (Figure 3). To note, the connection of MD2-SBro and the GST-tag is a linker Asp-Asp-Asp-Asp-Lys fragment, which is a cleavage site for enterokinase. The presence of this site allows the production of MD2-SBro free from the tag via digestion by the enterokinase. While it is unlikely that GST modulates or diminishes the activity, it is unclear if the linker participated in the activity. Given the linker is quite short (< 10 amino acids) and located far from the active sites, it is unlikely that the linker affects the activity; nevertheless, this remains to be experimentally confirmed.

### 3.4. Enzymatic Activity and Kinetic Parameters

**Figure 4:**
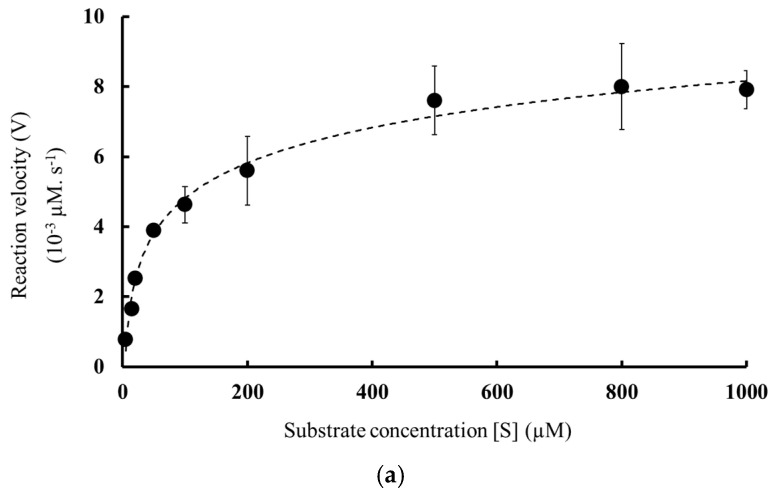

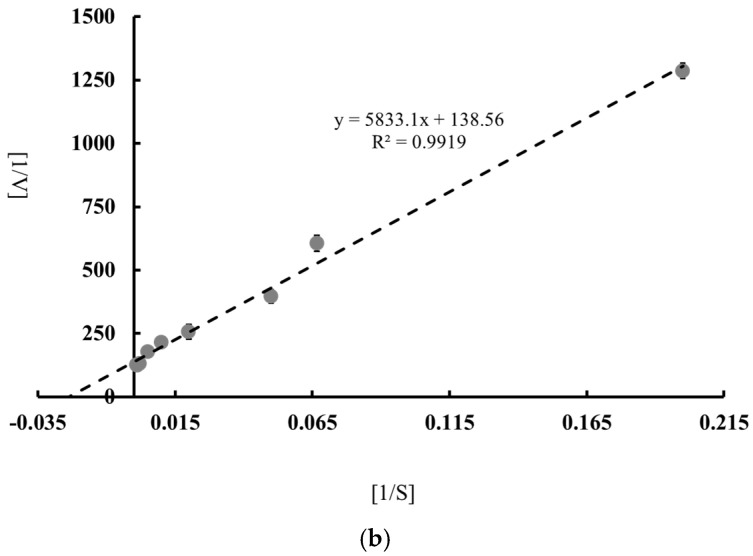
(**a**) Michaelis–Menten curve and (**b**) Lineweaver–Burk double reciprocal plot of MD2-SBro.

Further, to estimate the kinetic parameters V_max_, K_m_, *k*_cat_, and catalytic efficiency (*k*_cat_/K_m_), additional tests were carried out at 37 °C and pH 5.0 by varying substrate concentration. Figure 4 shows the Michaelis–Menten curve and Lineweaver–Burk plot used for the basis of the kinetic parameter’s calculation. Accordingly, calculated kinetic parameters of MD2-SBro were shown in Table 3, with the catalytic efficiency of 4.75 ± 0.23 × 10^−3^ µM^−1^ s^−1^.

### 3.5. Optimum Temperature and pH

**Figure 5:**
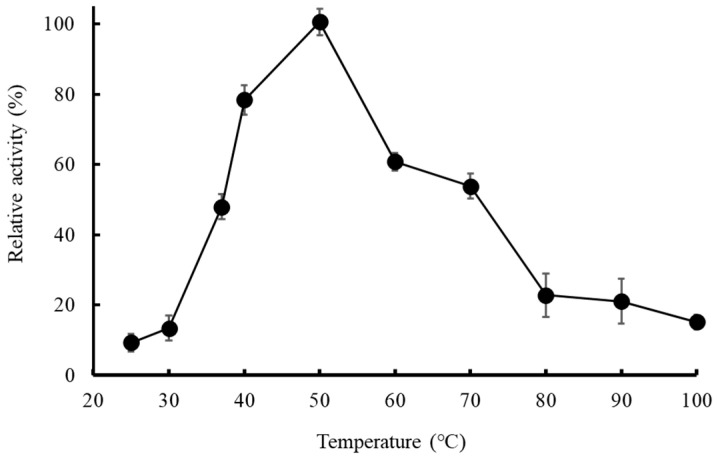
Temperature-dependent activities of the purified MD2-SBro. The highest activity at 50 °C (19.77 × 10^−3^ U/mg) was adjusted as 100%.

The optimum temperature of enzymatic activity was identified by carrying out activity assays at pH 8.0 and varying temperatures from 25–80 °C. The only initial values of enzyme activity were considered to minimize the influence of activity loss due to irreversible denaturation of protein [34]. As demonstrated in Figure 5, the optimum temperature of MD2-SBro was 50 °C.

**Figure 6:**
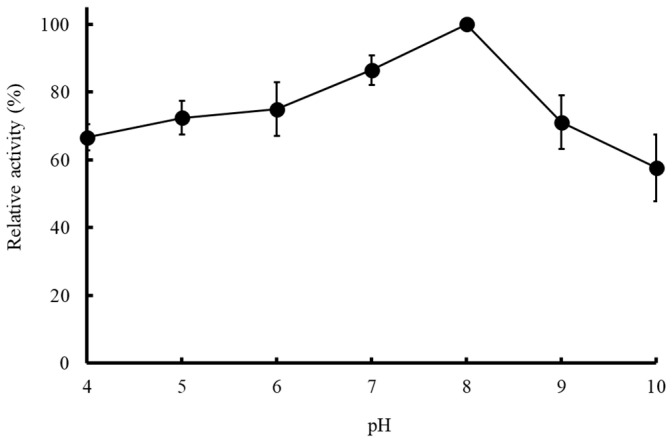
The pH-dependent activities of the purified MD2-SBro. The highest activity at pH 8.0 (3.56 × 10^−3^ U/mg) was adjusted as 100%.

Meanwhile, Figure 6 showed the pH-dependent activity of MD2-SBro, which was measured with pH 4.0–10.0. The data demonstrated that the MD2-SBro was active over a relatively wide pH range, and the highest activity towards the substrate was observed at pH 8.0.

### 3.6. Effect of EDTA and Metal Ions

As demonstrated in Table 4, the activity of MD2-SBro was decreased to about 17% in the presence of EDTA. In addition, the effect of different types of divalent ions on the catalytic activity of MD2-SBro was found to be varied. The addition of Mg^2+^, Ni^2+^, and Ca^2+^ ions increased the catalytic activity of MD2-SBro. Meanwhile, the addition of Zn^2+^ or Cu^2+^ metal ions decreased the activity of MD2-SBro. The reduction by Zn^2+^ was 41% for MD2-SBro. Meanwhile, Cu^2+^ decreased the activity of MD2-SBro by 22%.

### 3.7. Antioxidant Activity

**Figure 7:**
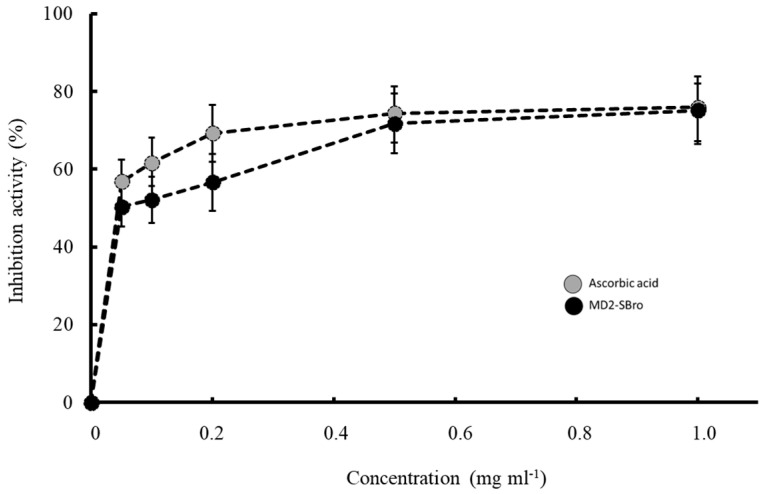
Inhibition of DPPH radical in the presence of different concentrations of ascorbic acid (control) and MD2-SBro.

The antioxidant activity of tested samples was conducted by DPPH assay, which is one of the most stable free radicals and is frequently used to evaluate radical scavengers in many types of samples [35]. The antioxidant activity of MD2-SBro was determined through their ability to scavenge the DPPH radical and therefore inhibit the formation of a radical form of DPPH. Figure 7 shows that the ability of MD2-SBro to inhibit the formation of a radical form of DPPH was in a concentration-dependent fashion. This is similar to the ability of ascorbic acid as a positive control. This indicated that MD2-SBro exhibited antioxidant activity through scavenging DPPH radicals. The calculated IC_50_ value for MD2-SBro to inhibit the DPPH radical formation was 0.022 mg mL^−1^, slightly higher than the IC_50_ value for ascorbic acid (0.018 mg mL^−1^).

## 4. Discussion

The common challenge in the production of recombinant bromelain is dealing with expressibility issues upon heterologous expression. One factor that might account for this issue is the variations and incompatibility of the codon profile of the target gene with the host cells to express [36,37]. Our previous approach using a codon-optimized gene successfully produced recombinant medium-sized bromelain from MD2-pineapple (MD2-MBro, size of 38 kDa) [1]. Accordingly, a similar approach might also work for the heterologous expression of MD2-SBro. In the current study, the gene encoding MD2-SBro was optimized to meet the requirement for expression under *E. coli* host cells. The value of GC content for the optimized MD2-SBro gene was in the range of favorable GC content for *E. coli* host cells [38]. In addition, the final CAI of the MD2-SBro gene was also re-adjusted to be compatible with the codon preferences of the host. Meanwhile, AT-rich regions were removed in the new sequence to avoid premature translational termination [39]. To note, the optimization did not affect the translated amino acid sequence as it dealt only with the changes in the DNA sequences.

Figure 1 confirmed that MD2-SBro was successfully over-expressed in *E. coli* cells. This indicated that the optimized gene of MD2-SBro enables this gene to be compatible with the *E. coli* system for expression. However, MD2-SBro was expressed in an insoluble form (inclusion body), despite originating from the modified gene. Notably, several expression conditions were attempted for MD2-SBro, particularly by varying the incubation temperature and period. Nevertheless, all these conditions resulted in insoluble expressed protein (data not shown). Recently, Bhatwa et al. [40] implied that the formation of an inclusion body is associated with the genetic regulation upon transcription and translation. Codon optimization is essentially related to gene expression through transcription regulation [41]. Nevertheless, as MD2-SBro is expressed as an inclusion body, this indicated that the codon optimization did not sufficiently contribute to the solubility of MD2-SBro upon heterologous expression. This is in agreement with an earlier report that implied the use of codon-optimized sequences did not affect the quality of the inclusion bodies obtained [36]. The formation of the inclusion body of MD2-SBro might be due to post-translational events, particularly the misfolding of protein. Obeng et al. [42] and Razali et al. [43] implied that the production of recombinants in *E. coli* is often challenged by its insolubility due to folding issues. In addition, the high expression level of the expressed protein might also contribute to the formation of the inclusion body due to the high concentrations of folding intermediates, which are prone to clump and aggregated [44,45,46]. To note, the standard for the heterologous protein to be considered as highly expressed is varied. In this study, 14 mg of MD2-SBro was expressed from 1 L of culture, which sounds to be not a high-level expression. Nevertheless, this expression level is much higher than other recombinant bromelain expressions reported by Iffah et al. [47], Amid et al. [19], and George et al. [48]. To note, all these bromelains were expressed from the nonoptimized gene. Interestingly, MD2-MBro was expressed at a higher level (20 mg/L culture) than MD2-SBro [1] in a fully soluble form. The discrepancy might be due to the differences in the physicochemical properties between both proteins. Bhatwa et al. [40] reported that the formation of the inclusion body of expressed protein is also governed by the structural and physicochemical properties of the proteins themselves. These features include the molecular weight, the number of adjacent hydrophobic residues, and the regions of low complexity.

Of note, MD2-SBro was expressed in a fusion form with a GST-tag. The tag was known not only to assist the purification process, but was also able to enhance solubility. Nevertheless, MD2-SBro remains expressed as an insoluble form or inclusion body. Inclusion bodies were classically considered amorphous types of protein aggregates devoid of any structural regularity [49]. Costa et al. [50] reported that the GST-tag theoretically acts for affinity and solubility enhancer purposes. Nevertheless, Boisselier et al. [51] reported that despite the high solubility of GST, not all GST-tagged fusion proteins are solubilizable. This is possibly due to the uniqueness of each protein, particularly in its hydrophobicity degree. Young et al. [52] reported that hydrophobic regions of the proteins might lead to unspecific interaction, which further caused the aggregation and became an inclusion body.

Following the solubilization of the insoluble protein of MD2-SBro using urea, the protein was successfully refolded. The use of urea in this study is considered the most common and conventional way to solubilize recombinant proteins from the inclusion body upon expression from *E. coli* cells [53]. The refolded protein was found to be in a soluble form (Figure 1), and this showed that the solubilization and refolding process of MD2-SBro inclusion body proteins succeeded. In addition, it also proved that the efficiency of these two steps is high. However, in some cases, the renaturation yields may be limited by the accumulation of inactive misfolded species and aggregates [54,55].

However, the purification yield of MD2-SBro protein after the single-step purification in this study is considered lower than other recombinant bromelains [1,19]. This is probably due to the low recovery of the protein solubilization or refolding, although the expression level was high. Nevertheless, this value is higher than purified recombinant bromelain in the study of Arshad et al. [47] and Bala et al. [56]. Unfortunately, previous studies that involved recombinant bromelain by Muntari et al. [18] and George et al. [48] did not report the purification yield for comparison.

In addition, the specific activity of MD2-SBro was only 3.56 ± 0.08 U/mg, which was considerably lower than MD2-MBro [1]. Nevertheless, as shown in Table 2, the purification yield of MD2-SBro was 80%, comparable to that of MD2-MBro reported earlier [1]. A high purification fold for MD2-SBro was speculated due to the use of GST affinity chromatography, which was reported to be very specific. MD2-MBro, in contrast, was expressed in a His-tag form, which has less specificity during affinity chromatography. Robichon et al. [57] reported that many indigenous *E. coli* proteins display high affinity to divalent nickel or cobalt ions, mainly due to the presence of clustered histidine residues or biologically relevant metal-binding sites. These indigenous proteins lead to low specificity of Ni-NTA chromatography compared to GST affinity chromatography. In this study, the GST-tag was not removed from MD2-SBro as there were no reports that GST exhibited proteolytic activity that would interfere with the MD2-SBro activity. Earlier, we also demonstrated that the large-sized tag of thioredoxin did not interfere with the activity of recombinant MD2-SBro [1]. In addition, the use of protease to cleave the linker between GST-tag and MD2-SBro is concerning due to the possibility of unspecific cleavage of MD2-SBro by the protease. As shown in Figure 3, it is evident that GST has no effect on the cleavage of *p*NP by MD2-SBro, as indicated by no changes in the yellow color of the cocktail upon the addition of free GST protein at different concentrations. Free GST protease has also demonstrated no proteolytic activity against the substrate due to no yellow color formation. Accordingly, it is suggested that MD2-SBro mainly generates the activity observed in this study.

The calculated kinetic parameters of MD2-SBro (Table 3) clearly demonstrated that catalytic efficiency (*k*_cat_/K_m_) of MD2-SBro was more than 11-fold lower than that of MD2-MBro, as reported earlier [1]. The differences might be due to their structural discrepancies or the presence of a GST-tag. Earlier, the model structure of MD2-SBro revealed that the Cys-His active site position of MD2-SBro was found to be inappropriate for catalysis. In addition, the substrate-binding pocket of MD2-SBro was found to be less hydrophobic than that of MD2-MBro [16]. This structural feature might account for the low activity of MD2-SBro. Notably, the catalytic efficiency of MD2-SBro was also much lower than the other bromelains, ranging from 17.86–52.53 µM^−1^ s^−1^, depending on the type of bromelain and substrate used in the assay [19,29].

Further, the optimum temperature of MD2-SBro, which was observed at 50 °C, suggested that the MD2-SBro behaves as a mesophilic protein, where it optimally worked at moderate temperature. This optimum temperature is similar to that of MD2-MBro [1]. Bala et al. [58] reported that non-recombinant bromelain from fruit or stem pineapple generally exhibited optimum activity at a temperature ranging from 40–70 °C. Nevertheless, Corzo et al. [59] discovered that the optimum temperature for the catalytic activity of bromelain was different depending on the substrate. Of note, the optimum temperature of MD2-SBro was higher than favorable growth temperatures in pineapple farms (18–32 °C) [60]. Nevertheless, it remains to be investigated if the optimum temperature of this protein is associated with its biological roles in the pineapple fruit. Meanwhile, the optimum pH of MD2-SBro was found to be 8.0, which is higher than that of MD2-MBro (pH 6.0), as reported earlier [1]. Nevertheless, the optimum pH of MD2-SBro remains in the range of the common optimum pH for stem and fruit bromelain, which was reported to be between 6.0–8.5 [14,61,62,63,64,65]. Interestingly, in the range of pH 4.0–7.0 and pH 9.0–10.0, MD2-SBro remains active with residual activity of > 60% (Figure 5). Of note, the pH optimum of bromelains was reportedly different by many authors due to the use of different substrates [59,66]. Nevertheless, Vernet et al. [67] previously proposed that an acidic pH is more favorable for bromelain if it triggers the pro-domain from the active site. Consequently, this makes the cleavage site within the pro-domain loop accessible to the active site, and the enzyme becomes activated [68]. Notably, MD2-SBro is a small protein with no pro-domain segment [16]. Accordingly, acidic pH is not necessarily required for activating MD2-SBro through the detachment of the pro-domain segment. The absolute specific activity values of MD2-SBro at its optimum temperature (50 °C) and pH (8.0) were 19.77 × 10^−3^ and 3.56 × 10^−3^ U/mg, respectively. These values are lower than the specific activity of MD2-MBro at 50 °C (10.22 × 10^−2^ U/mg) or at pH 8.0 (6.13 × 10^−2^ U/mg).

It is interesting to find that the activity of MD2-SBro was decreased by EDTA, which is in good agreement with the previous study reported by Hidayani et al. [28]. The negative effect of EDTA on the activity of MD2-SBro is due to the chelating of metal ions in the catalytic site of the enzyme by EDTA and altering of the structure, as was also proposed by Hidayani et al. [28]. Nevertheless, the identities of the metal ions required for the enzyme activity are yet to be investigated. A reasonable way to identify the metal binding site of this protein is through co-crystallization with the metal ions. The putative residues for metal ion coordination are further confirmed through the mutagenesis approach.

Meanwhile, the increasing activity of MD2-SBro due to the presence of different types of divalent ions (Mg^2+^, Ni^2+^, and Ca^2+^) is similar to previous studies of bromelain [1,30,69,70,71,72]. The increase of catalytic activity of MD2-SBro in the presence of Mg^2+^, Ni^2+^, and Ca^2+^ were 203%, 118%, and 134%, respectively, higher than that in the absence of any metal ions. The observed effects of Ca^2+^ on bromelain activity are in good agreement with the earlier reports [73,74,75] that calcium ions promote bromelain activity by stabilizing the secondary structure of an enzyme. According to Fadhilah et al. [69], the addition of Mg^2+^ also aids in maintaining the conformation of bromelain, which is important in the occurrence of catalytic activity. Unfortunately, so far, no detailed study on the effect of Ni^2+^ on bromelain activity is available.

Figure 7 also showed a reduction of MD2-SBro activity by adding Zn^2+^ or Cu^2+^ metal ions. Similar results were also reported for the effect of these two ions on bromelain [30,70,72,76]. The effects of Cu^2+^ on bromelain activity corroborate the earlier observations [1,72,74,76,77], which implied that copper ions inhibit the bromelain activity by forming a coordination bond with a catalytic sulfhydryl group.

The interesting bioactivity of bromelain is its antioxidant activity, which remains unknown as to whether it is associated with its catalytic activity. Some reports have clearly demonstrated the antioxidant activity of non-recombinant bromelain against DPPH radical or lipid peroxidation inhibition [78,79,80,81]. Nevertheless, there has been no report so far on the antioxidant activity of bromelain produced through the recombinant approach. Figure 7 clearly shows that MD2-SBro could scavenge DPPH as one of the phenotypical antioxidant activities. It is unclear how bromelain scavenges the DPPH radical. However, it might be due to the antioxidant properties of individual amino acids of MD2-SBro. Udenigwe et al. [82] reported that sulfur-containing (SCAA), acidic, and hydrophobic amino acids had strong positive effects on scavenging of 2,2-diphenyl-1-picrylhydrazyl (DPPH). All these residues are found in the MD2-SBro sequence. Chakraborty et al. [83] highlighted that the antioxidant activity of bromelain put this enzyme as a potential food-based bioactive compound for various pharmaceutical applications. Ataide et al. [81] reported that bromelain’s antioxidant activity might be associated with bromelain’s activity in the modulation of the inflammatory system and skin debridement properties. Of note, most of the antioxidant activity on bromelain used an unpurified (crude) form of bromelain, which leads to a possibility of bias by the antioxidant activity generated by the contamination. The ability of MD2-SBro to scavenge DPPH radically indicated that a single cysteine protease indeed exhibited antioxidant activity. The IC_50_ values of MD2-SBro to scavenge DPPH radical were found to be higher than that of crude bromelain reported by Abbas et al. [80], but lower than that reported by Saptarini et al. [78] and Huang et al. [79]. This indicated that each cysteine protease bromelain possesses unique antioxidant properties.

Of note, the bromelain gene studied in this study originated from MD2 pineapple. This is due to the availability of its whole genome sequence. In addition, MD2 pineapple is currently also the major pineapple variant planted in Malaysia [84]. Therefore, the current study should provide insight into the promising bioactivity of bromelain from MD2 pineapple for further studies, and scale-up productions using a heterologous expression approach.

## 5. Conclusions

This study demonstrated the success of the production of MD2-SBro, one of the enzyme-based bioactive compounds from MD2-pineapple, using a heterologous expression system using *E. coli* host cells. While recombinant MD2-SBro was produced in the inclusion body, this protein could be solubilized and refolded to form active bromelain. Intriguingly, MD2-SBro is proven to be active, albeit with little specific activity and low catalytic efficiency. The pH and temperature optimum and the metal-ion dependency of this protein were found to behave uniquely compared to other bromelains. Interestingly, the antioxidant activity of MD2-SBro is remarkably higher and close to the well-known antioxidant of ascorbic acid. Future studies in these characterizations may lead to the expansion of small-sized bromelain applications. The expressibility of MD2-SBro in *E. coli* host cells is an important milestone for the production of this protein for further studies and applications as a promising bioactive compound.

## Figures and Tables

**Table 1:** Gene optimization of the MD2-SBro gene.

Parameters	Original MD2-SBro Gene	Optimized MD2-SBro Gene
GC content	44.51%	53.34%
Codon adaptation index (CAI)	0.40	0.95

**Table 2:** Purification profile of MD2-SBro indicating its activity and yield.

Steps	Volume (mL)	Total Protein (mg)	Total Activity (U) *	Specific Activity (U/mg) *	Yield (%)	Purification (Fold)
Cell lysate	70 ± 2.80	510 ± 12	62.30 ± 5.64	0.12 ± 0.002	100	1.0
Glutathione S-transferase (GST) affinity chromatography	22 ± 1.38	14 ± 1.32	49.84 ± 3.17	3.56 ± 0.08	80	42.72

* measured at 37 °C, pH 8.0.

**Table 3:** Kinetic parameters of MD2-SBro in comparison to MD2-MBro.

Proteins	V_max_ (10^−3^ µM s^−1^)	K_m_ (µM)	*k*_cat_ (s^−1^)	*k*_cat_/K_m_ (10^−3^ µM^−1^ s^−1^)	Ref
MD2-SBro	7.20 ± 0.52	42.1 ± 3.81	0.20 ± 0.008	4.75 ± 0.23	This study
MD2-MBro	15 ± 0.5	34.24 ± 1.02	1.93 ± 0.05	56.37 ± 2.08	[1]

**Table 4:** Relative activity of MD2-SBro in the presence of various metal ions and EDTA.

Metal Ions	Relative Activity (%)
Control	100 ± 5.01
MgCl_2_	178.32 ± 7.54
CaCl_2_	121.40 ± 8.71
NiCl_2_	114.31 ± 10.32
CuCl_2_	78.09 ± 3.21
ZnCl_2_	47.36 ± 3.98
EDTA	17.58 ± 1.07

Note: Control refers to the activity with no metal ions or EDTA.

## Data Availability

Not applicable.
